# Bicarbonate-Dependent Secretion and Proteolytic Processing of Recombinant Myocilin

**DOI:** 10.1371/journal.pone.0054385

**Published:** 2013-01-16

**Authors:** José-Daniel Aroca-Aguilar, Francisco Martínez-Redondo, Alba Martín-Gil, Jesús Pintor, Miguel Coca-Prados, Julio Escribano

**Affiliations:** 1 Laboratorio de Genética Molecular Humana, Facultad de Medicina/Instituto de Investigación en Discapacidades Neurológicas (IDINE), Universidad de Castilla-La Mancha, Albacete, Spain; 2 Departamento de Bioquímica y Biología Molecular IV, E.U. Óptica, Universidad Complutense de Madrid, Madrid, Spain; 3 Fundación de Investigación Oftalmológica Instituto Oftalmológico Fernández-Vega, Oviedo, Spain; 4 Department of Ophthalmology and Visual Science, Yale University School of Medicine, New Haven, Connecticut, United States of America; Ottawa Hospital Research Institute, Canada

## Abstract

Myocilin is an extracellular glycoprotein of poorly understood function. Mutations of this protein are involved in glaucoma, an optic neuropathy characterized by a progressive and irreversible visual loss and frequently associated with elevated intraocular pressure. We previously showed that recombinant myocilin undergoes an intracellular proteolytic processing by calpain II which cleaves the central region of the protein, releasing one N- and one C-terminal fragment. Myocilin cleavage is reduced by glaucoma mutations and it has been proposed to participate in intraocular pressure modulation. To identify possible factors regulating the proteolytic processing of recombinant myocilin, we used a cellular model in which we analyzed how different culture medium parameters (i.e., culture time, cell density, pH, bicarbonate concentration, etc.) affect the presence of the extracellular C-terminal fragment. Extracellular bicarbonate depletion associated with culture medium acidification produced a reversible intracellular accumulation of full-length recombinant myocilin and incremented its intracellular proteolytic processing, raising the extracellular C-terminal fragment percentage. It was also determined that myocilin intracellular accumulation depends on its N-terminal region. These data suggest that aqueous humor bicarbonate variations could also modulate the secretion and cleavage of myocilin present in ocular tissues.

## Introduction

Myocilin is a 54 kDa extracellular glycoprotein that belongs to the olfactomedin family of proteins. Mutations in the *MYOCILIN* gene (*MYOC*) are involved in different types of glaucoma [1]–[3]. This protein is expressed in muscular and ocular tissues such as iris, ciliary body (CB) and trabecular meshwork (TM) and is especially abundant in aqueous humor (AH) [4], [5], where it forms large molecular homo- and hetero-aggregates [6]–[8]. Myocilin is a modularly structured protein consisting of three independently folded functional domains encoded by three exons. Exon 1 encodes the N-terminal domain containing two coiled coils and one leucine zipper motif and is involved in myocilin self-aggregation. Exon 2 encodes the central linker domain [9] which is cleaved by calpain II [10], splitting the N- and C-terminal domains, and exon 3 encodes the olfactomedin-like globular domain. In spite of the considerable efforts made to unravel myocilin’s biology, both normal and pathological functions of this protein remain largely unknown. Glaucoma myocilin mutations impair the secretion [11], [12] and proteolytic processing [9] of the protein. Furthermore, co-expression of wild-type and glaucoma mutant myocilin leads to both reduced myocilin secretion and processing [13]. Similarly, the functional meaning of the proteolytic processing is currently poorly understood, although we reported that it affects myocilin molecular homo- [14] and hetero-interactions with other extracellular matrix proteins, such as hevin, SPARC, fibronectin and laminin [15].

Regarding the molecular mechanisms involved in myocilin proteolytic processing, we previously observed that myocilin cleavage increased over time when the recombinant protein is expressed in cell lines (i.e., HEK-293T) [10], [13]. In an effort to characterize the molecular mechanisms involved in this process, we studied how different culture parameters and factors (e.g., culture time, pH, bicarbonate concentration, etc.) affect myocilin's cleavage. We show that extracellular bicarbonate exhaustion leads to intracellular accumulation of full-length myocilin and increases its proteolytic processing. We propose that AH bicarbonate variations could regulate secretion and cleavage of myocilin present in ocular tissues.

## Materials and Methods

### Recombinant Myocilin Expression in Cell Cultures

The human embryonic kidney 293T cell line (HEK-293T) was obtained from the ATCC (American Type Culture Collection) and the human ocular cell line hCM was established from the primary culture of ciliary muscle cells of a 26-year-old male (cadaver) by viral transformation [10]. hCM cells stably expressing recombinant myocilin were obtained by selection of transfected cells with 1.5 mg/ml G-418 (Promega) for 7 days, and analyzed in the following passages.The two cell lines were maintained in Dulbecco’s modified Eagle’s medium (DMEM) supplemented with 10% fetal bovine serum (FBS) and antibiotics (Normocin, Invitrogen) at 37°C in a fully humidified 5% CO_2_ atmosphere. All transfections were carried out with 200 ng of cDNAs encoding wild-type myocilin [9], N-terminal or C-terminal myocilin fragments [10], using the Superfect Transfection Reagent (Qiagen), according to the manufacturer’s instructions. cDNAs were cloned into the pcDNA3.1-myc-His vector. After transfection cells were cultured either under the earlier described conditions or in serum- and bicarbonate-free DMEM (Gibco) buffered with HEPES (75 mM, pH 7.1). In the latter case, the cells were incubated at 37°C in a fully humidified incubator with room CO_2_ atmosphere.

To remove cellular debris from the collected culture medium, samples were centrifuged at 5000 g for 5 min. The supernatant (culture medium) was stored at −80°C until used. Adhered cells were washed twice with 1 ml of Dulbecco's phosphate-buffered saline (DPBS; 150 mM NaCl, 3 mM KCl, 1 mM KH_2_PO_4_, 6 mM Na_2_HPO_4_, 0.5 mM MgCl_2_, 1 mM CaCl_2_, pH 7.2), followed by the addition of 200 µl of lysis buffer [50 mM Tris-HCl, pH 7.4; 150 mM NaCl; 1% (v/v) IGEPAL CA-630 (Sigma-Aldrich); 1 mM EDTA; 1 mM PMSF, 1 μg/ml Leupeptin; 1 mM Na_3_VO_4_ and 1 mM NaF] containing protease inhibitors. Collected cells were vortexed for 30 s at maximum speed, incubated on ice for 30 min and sonicated for 10 s (cycle, 0.5 s). Cell lysates were stored at −80°C until used. The efficiency of transfection was estimated in cells transfected with a cDNA construct encoding GFP by counting the number of GFP-positive cells in a total of 10^3^ cells in four randomly selected fields per dish. In all the experiments extracellular LDH was analyzed by Western blot to confirm that the different treatments did not produce cell death.

### Western Blotting and Antibodies

For Western blot analysis, aliquots of culture medium and cell lysates were treated with loading buffer containing β-mercaptoethanol, boiled for 5 min, and fractionated by 10% polyacrylamide gel electrophoresis in the presence of SDS [16], using the Mini-PROTEAN III gel electrophoresis system (BioRad). To ensure that the same amount of protein was loaded in each lane the protein content was determined by the Bicinchoninic Acid Protein Assay Kit (Thermo Scientific), following the manufacturer’s recommendations. Routinely, cell lysates and culture medium aliquots containing 20 and 80 μg of total protein, respectively, were analyzed. Aliquots from serum- and bicarbonate-free DMEM contained 2 μg of total protein. When different culture medium volumes were used the amount of total protein was adjusted proportionally. Gels were subsequently transferred onto Hybond ECL nitrocellulose membranes (Amersham) for immunodetection. Ponceau S (Panreac) staining of blots prior to antibody incubation was performed to ensure the integrity of samples and that equal amounts of sample were analyzed [17]. A commercial mouse monoclonal anti-myc antibody (sc-40, Santa Cruz) or anti-HA (sc-7392, Santa Cruz) was used as primary antibody, at the 1∶500 dilution. A secondary anti-mouse IgG horse-radish peroxidase-conjugated antibody (#32430, Thermo Scientific) was diluted 1∶1000. As an additional control of sample loading and cell integrity lactate dehydrogenase (LDH) was detected in cell extracts and culture media using a goat anti-LDH antibody (AB1222, Chemicon, diluted 1∶5000) and anti-goat IgG horse-radish peroxidase-conjugated antibody (sc-2033, Santa Cruz, diluted 1∶2000). Chemiluminescence analysis was performed with Supersignal Dura Western Blot reagents (Thermo Scientific) using the LAS3000-mini (Fujifilm, Tokyo, Japan) detector. Densitometry for protein band quantification was performed using Quantity One 4.1 analysis software (BioRad) in at least two independent experiments performed in triplicate. Statistical comparison between groups was performed using one- or two-way analysis of variance (ANOVA), followed in some experiments by Tukey’s multiple comparison test, and calculated with the SigmaStat 2.0 software (SPSS Science).

### Effect on Recombinant Myocilin Production of Culture Time, Cell Density, Nitric Oxide Activators or Inhibitors, Culture Medium pH and Sodium Bicarbonate

To check the possible effect of nitric oxide, cells transiently expressing recombinant myocilin were treated with each of the following compounds for 18 h: the nitric oxide activators diethylamine NONOate (10 μM) (Sigma-Aldrich) and S-Nitrosoglutathione (100 μM) (GSNO, Sigma-Adrich); the nitric oxide inhibitor Nω-nitro-L-arginine methyl ester (200 μM) (L-NAME, Sigma-Aldrich); and the antioxidants L-Glutathione reduced (500 μM) (GSH, Sigma-Aldrich) and Ebselen (50 μM) (Sigma-Aldrich). To assess the effect of pH and sodium bicarbonate on myocilin processing the cells were incubated with 0,01% (w/v) L-ascorbic acid (Sigma-Aldrich), 5 mM N-acetyl-cysteine (Sigma-Aldrich), which is also an antioxidant, 20 mM NaOH (Panreac), 32 mM sodium piruvate (Cambrex) or 20 mM sodium bicarbonate (Panreac). Stock solutions of the different compounds, except Ebselen, were prepared in PBS. Ebselen was dissolved in DMSO and diluted with PBS to a final concentration of less than 1‰ DMSO in cell culture. Sterile filtration of the compounds was achieved using 0.2-μm filter devices (VWR). Working solutions were prepared immediately before use and kept on ice until added to culture media.

To identify the possible factors responsible for myocilin cleavage activation, first we tested the role of reactive oxygen species (ROS) and free radicals because these metabolic compounds are known to increase over time in the culture medium. Nitric oxide is a reactive free radical; therefore cell cultures transiently expressing recombinant myocilin were treated with nitric oxide activators nonoate and GSNO, nitric oxide inhibitor L-NAME, or antioxidants GSH and ebselen. The culture conditions were selected to obtain approximately 50% cleaved extracellular myocilin (400000 HEK-293T cells in 300 µl of medium, cultured for 18 h). None of these treatments affected the proportion of the C-terminal fragment, which shows that a direct implication of ROS and free radicals in myocilin cleavage is unlikely.

## Results

### Myocilin Proteolytic Processing Dependence on Cell Density and Culture Time

**Figure 1:**
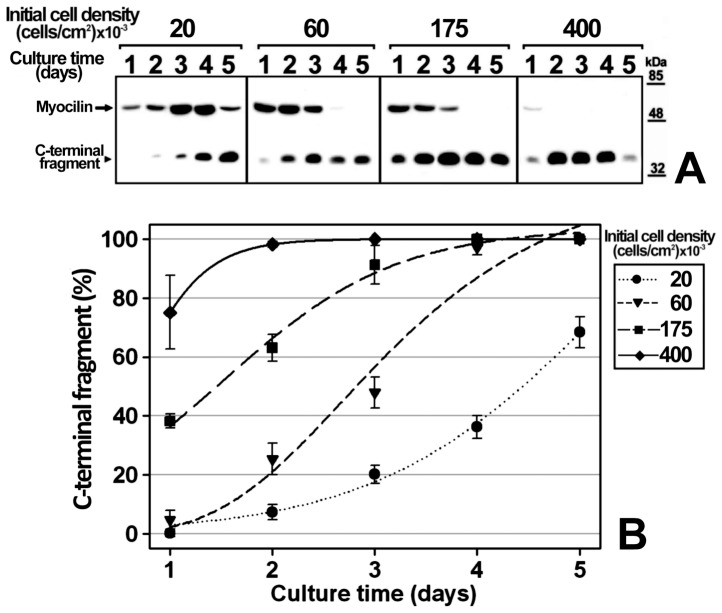
Effect of culture time and initial cell density on recombinant myocilin proteolytic processing. (A) HEK-293T cells were seeded in 6-well plates at the indicated cell densities. Cells were transfected with a cDNA construct encoding myocilin-myc as described in Materials and Methods. After transfection, they were cultured in DMEM containing 10% FBS for 5 days and the culture media were substituted daily by equal volumes of fresh medium. Extracellular recombinant myocilin was analyzed by 10% polyacrylamide SDS-PAGE and Western blot using an anti-myc monoclonal antibody (Santa Cruz). Equal amount of total protein was loaded into each well. The arrow and arrowhead indicate the position of full-length myocilin (55-kDa), and the 35-kDa C-terminal fragment, respectively. (B) Quantitation by densitometry of the C-terminal fragment detected in *A.* Values represent the percentage of extracellular myocilin C-terminal fragment expressed as: 100x*[C-terminal fragment/(C-terminal fragment+full length myocilin)]*. Error bars correspond to the SD of three independent experiments carried out in triplicate Two-way ANOVA showed an effect of culture time (*p*<0.001) and cell density (*p*<0.001), and a significant interaction between them (p<0.001).

**Figure 2:**
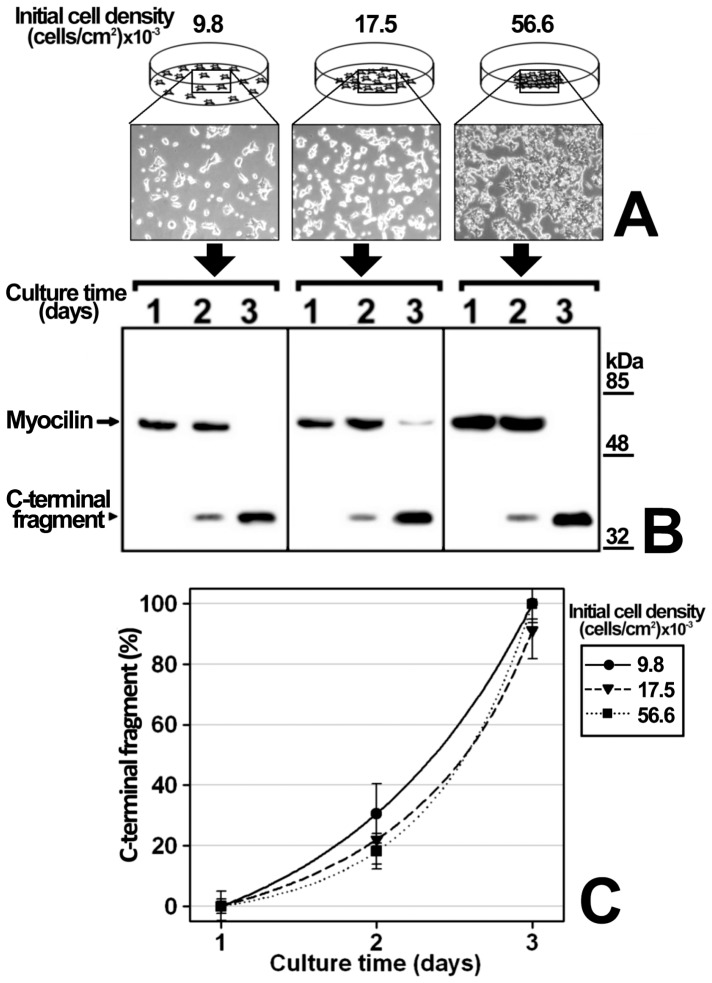
Influence of initial cell density in myocilin proteolytic processing. (A) HEK-293T cells (400000 cells/plate) were seeded in different areas of cell plates using circular frames to obtain the indicated cell densities. Magnification of phase contrast micrographs: 40X. Cells were transfected with a cDNA construct encoding myocilin-myc as described in Materials and Methods. After transfection, culture medium aliquots were collected every day. (B) Extracellular recombinant myocilin was analyzed by 10% polyacrylamide SDS-PAGE and Western blot using an anti-myc monoclonal antibody. Equal amount of total protein was loaded into each well. The horizontal arrow and arrowhead indicate the position of full-length myocilin (55-kDa), and the 35-kDa C-terminal fragment, respectively. (C) Quantitation by densitometry of the C-terminal fragment detected in *B.* Values represent the proportion of this myocilin fragment, expressed as indicated in Fig. 1B. Error bars correspond to the SD of three independent experiments carried out in triplicate. Two-ways ANOVA showed an effect of culture time (*p*<0.001) but not of cell density (*p*>0.1). No significant interaction between culture time and cell density was observed (p>0.1).

**Figure 3:**
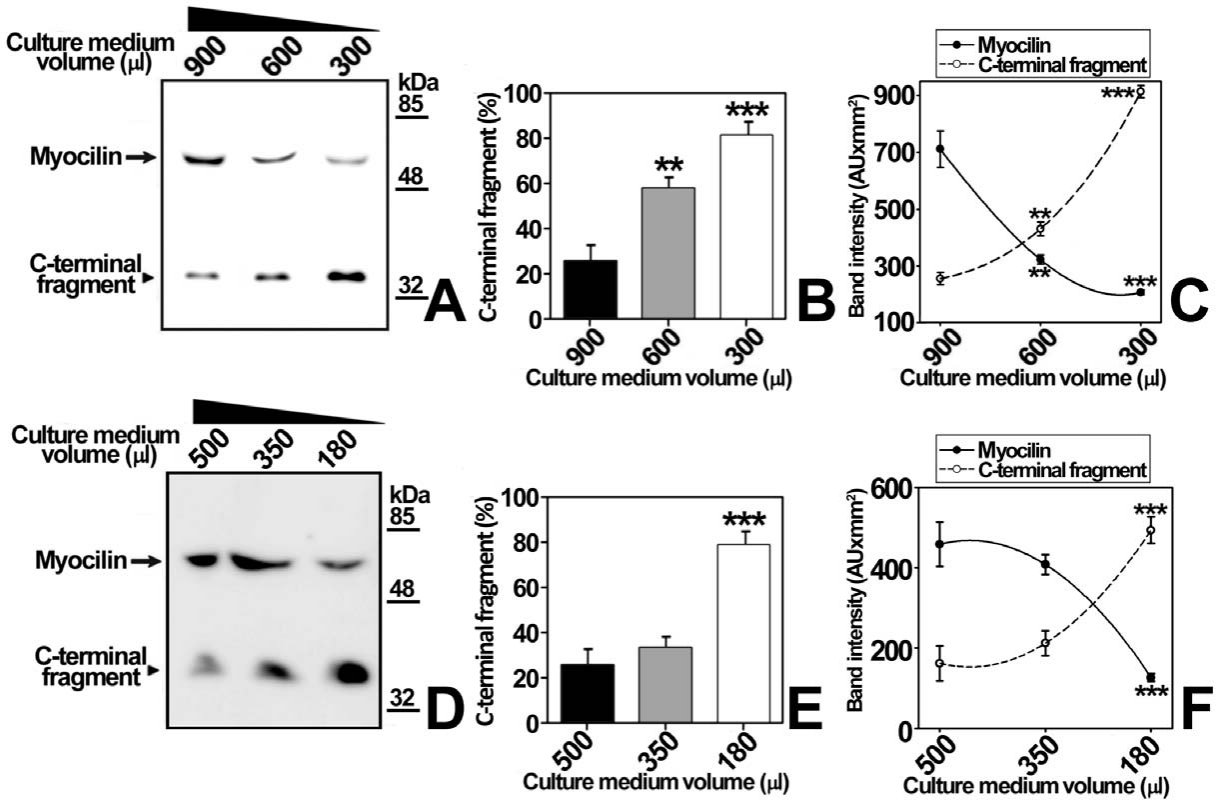
Influence of the culture medium volume in proteolytic myocilin processing. (A) HEK-293T cells (400000 cells/well) were transfected with a cDNA construct encoding myocilin-myc. After transfection, the indicated volumes of culture medium were added to each well and collected 48 h later. Extracellular recombinant myocilin was analyzed in normalized aliquots representing 5% of the total culture medium volume by 10% polyacrylamide SDS-PAGE and Western blot using an anti-myc monoclonal antibody. (B) Densitometric relative quantitation of the C-terminal fragment detected in *A.* Values represent the proportion of this myocilin fragment, expressed as indicated in Fig. 1B. (C) Densitometric quantitation of the full-length and C-terminal myocilin fragment detected in A. (D) The human ocular cell line hCM stably expressing recombinant myocilin-HA (400000 cells/well) were cultured with different volumes of culture medium as indicated in (A). (E) and (F) Recombinant myocilin secrected by hCM cells was quantitated as indicated in (B) and (C), respectively. Error bars correspond to the SD of two independent experiments carried out in triplicate. Asterisks indicate statistical significance compared to the first volume: p<0.01 (**); p<0.001 (***). One-way ANOVA followed by Tukey multiple-comparison test.

We previously reported that myocilin cleavage is culture time-dependent in the human 26HCMsv and HEK-293T cell lines [10], [13]; however, the mechanism involved in this effect has not yet been elucidated. To study myocilin proteolytic processing the recombinant protein was transiently expressed in HEK-293T cells and a time-course analysis of the extracellular C-terminal fragment at different initial cell densities was carried out. This cell line was selected for the study because it expresses efficiently high cleaved myocilin levels [9], [18]. The recombinant protein was fused to the myc epitope at the C-terminal end, and the culture medium was replaced daily and extracellular LDH was analyzed to confirm that there was no detectable cell death (data not shown). According to previous results, full-length myocilin and the C-terminal fragment were detected as 55 and 35 kDa bands, respectively, when we used an anti-myc antibody in Western immunoblots [9], [10]. At any given initial cell density the proportion of extracellular myocilin C-terminal fragment [C-terminal fragment/(C-terminal fragment+full length myocilin)] increased over time (Fig. 1). Furthermore, at any given culture time, the extracellular fragment proportion increased with the initial cell density. After 5 days of cell culture at the highest initial cell density the amount of the fragment decreased due to reduce cell viability, but all detected myocilin was cleaved. Based on these results, we hypothesized that cell-to-cell contacts and/or metabolic-dependent medium changes could lead to an increased extracellular C-terminal fragment proportion. In these experiments there was a significant interaction between culture time and cell density (p<0.001; two-way ANOVA). Therefore, to determine whether or not the proteolytic processing depends on cell density, we followed-up the time-course evolution of the extracellular myocilin fragment produced by a constant cell number seeded at different initial densities (9800, 17500 and 56600 cells/cm^2^) (Fig. 2A). In contrast to the previous experiment, the culture medium was not replaced until the end of the assay. The extracellular C-terminal fragment proportion increased significantly over time (p<0.001; two-way ANOVA) in the three cell densities assayed, progressing from 0% at day 1, to 95–100% after 3 days (Fig. 2B and C) and no significant differences were observed among cell densities (p>0.1; two-way ANOVA). Under these conditions there was not significant interaction between culture time and cell density (p>0.1; two-way ANOVA). These results indicate that myocilin proteolytic cleavage is independent of cell density and confirms that it depends on culture time. Since the composition and pH of the culture medium change over time as a result of cell metabolism, we reasoned that metabolic-induced medium changes could mediate myocilin processing. Consequently, in order to intensify the metabolic-induced culture medium composition change, we grew HEK-293T cells transiently expressing recombinant myocilin for 48 h at a constant number and cell density but in decreasing culture medium volumes (from 900 to 300 μl, Fig. 3A and B). As expected, the extracellular C-terminal fragment proportion increased inversely proportional to the culture medium volume (Fig. 3A and B). Moreover, the total amount of this fragment increased as the amount of extracellular full-length myocilin decreased (Fig. 3C), suggesting an extracellular volume-dependent activation of the proteolytic cleavage. Similar results were obtained when recombinant myocilin was stably expressed in the ocular human ciliary muscle cell line hCM (Fig. 3D, 3E and F), although smaller culture media volumes were required to obtain C-terminal fragment levels similar to those observed in HEK-293T cells. These results support that metabolic-induced changes in the chemical composition of the culture medium activate myocilin proteolysis, increasing the presence of the extracellular C-terminal fragment. According to this hypothesis, we anticipated that the extracellular myocilin fragment will increase in the presence of conditioned media from cultured cells grown at high cell densities. As expected, the proportion of extracellular myocilin C-terminal fragment increased from 60% to 75% when transfected cells were grown in the presence of conditioned medium instead of fresh culture medium (Fig. S1). Nevertheless, we also observed that the total amount of synthesized protein decreased in the conditioned media as a result of nutrient exhaustion.

### Identification of the Culture Medium Component Regulating Myocilin Processing

**Figure 4:**
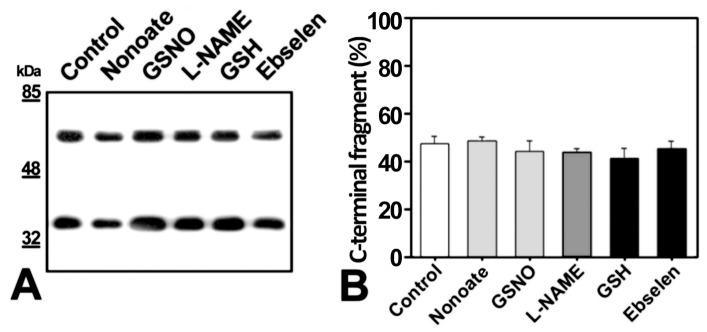
Effect of nitric oxide (NO) synthesis activators (Nonoate, GSNO) and inhibitors (L-NAME) and antioxidants agents (GSH, Ebselen) on myocilin proteolytic processing. (A) HEK-293T cells (500000 cells/plate) were transfected with a cDNA construct encoding myocilin-myc. After transfection the cells were treated with NO synthesis activators (Nonoate 10 μM or GSNO 100 μM), a nitric oxide synthesis inhibitor (L-NAME 200 μM), or antioxidizing agents (GSH 500 μM or Ebselen 50 μM). Culture media were collected 18 h later and the recombinant myocilin secreted to the culture medium was analyzed by 10% polyacrylamide SDS-PAGE and Western blot using an anti-myc monoclonal antibody. Equal amount of total protein was loaded into each well. (B) Quantitation by densitometry of the C-terminal fragment detected in *A.* Values represent the proportion of this myocilin fragment, expressed as indicated in Fig. 1B. Error bars correspond to the SD of two independent experiments carried out in triplicate.

To identify the possible factors responsible for myocilin cleavage activation, first we tested the role of reactive oxygen species (ROS) and free radicals because these metabolic compounds are known to increase over time in the culture medium. Nitric oxide is a reactive free radical; therefore cell cultures transiently expressing recombinant myocilin were treated with nitric oxide activators nonoate and GSNO, nitric oxide inhibitor L-NAME, or antioxidants GSH and ebselen (Fig. 4). The culture conditions were selected to obtain approximately 50% cleaved extracellular myocilin (400000 HEK-293T cells in 300 µl of medium, cultured for 18 h). None of these treatments affected the proportion of the C-terminal fragment, which shows that a direct implication of ROS and free radicals in myocilin cleavage is unlikely.

**Figure 5:**
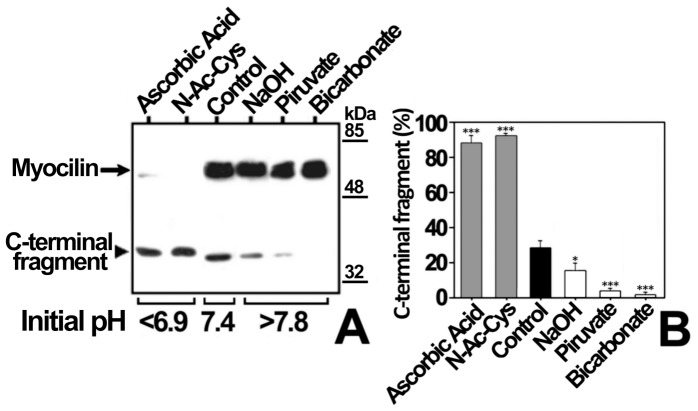
Effect of pH on myocilin proteolytic processing in bicarbonate-buffered medium. (A) HEK-293T cells (500000 cells/plate) were transfected with a cDNA construct encoding myocilin-myc. After transfection, cells were cultured in bicarbonate-buffered medium at different pH values adjusted with the compounds indicated over each lane. Culture media were collected 48 h later and the extracellular recombinant myocilin was analyzed by 10% polyacrylamide SDS-PAGE and Western blot using an anti-myc monoclonal antibody. Equal amount of total protein was loaded into each well. (B) Quantitation by densitometry of the C-terminal fragment detected in *A.* Values represent the proportion of this myocilin fragment, expressed as indicated in Fig. 1B. Error bars correspond to the SD of three independent experiments carried out in triplicate. Asterisks indicate statistical significance compared to control: p<0.05 (*); p<0.01 (**); p<0.001 (***). One-way ANOVA followed by Tukey multiple-comparison test.

**Figure 6:**
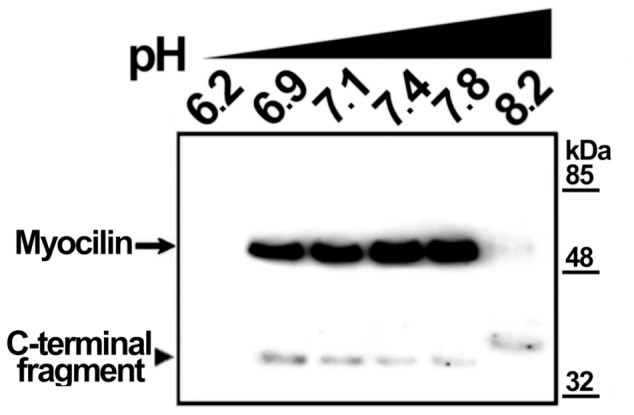
Effect of pH on myocilin proteolytic processing in bicarbonate-free medium. HEK-293T cells (200000 cells/plate) were transfected with a cDNA construct encoding myocilin-myc. After transfection, cells were cultured in bicarbonate-free medium, buffered at different pH values with HEPES. Cells were cultured in a humidified room air atmosphere at 37°C. Culture media were collected 48 h later and recombinant myocilin present in the culture medium was analyzed by 10% polyacrylamide SDS-PAGE and Western blot using an anti-myc monoclonal antibody. Equal amount of total protein was loaded into each well.

**Figure 7:**
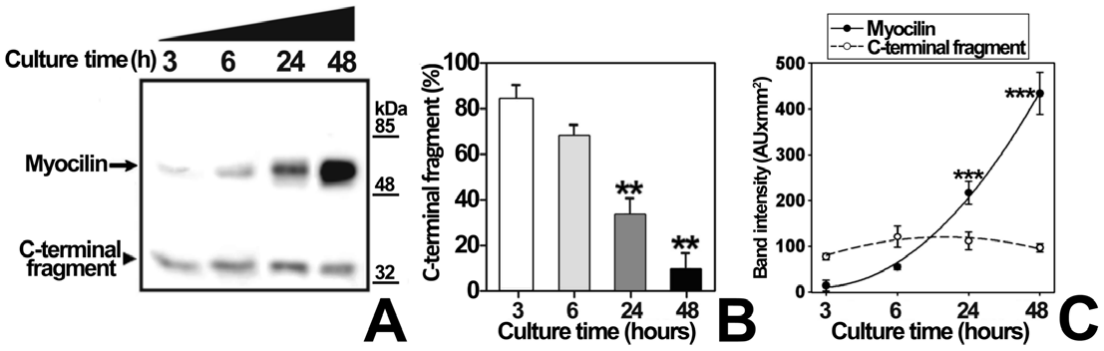
Time-course analysis of myocilin proteolytical processing in bicarbonate-free medium. (A) HEK-293T cells (200000 cells/plate) were transfected with a cDNA construct encoding myocilin-myc. After transfection cells were cultured in bicarbonate-free HEPES buffered medium, pH 7.1, as indicated in Fig. 6, and aliquots of the medium were collected at 3, 6, 24 and 48 h. Recombinant myocilin was detected in the culture medium by 10% polyacrylamide SDS-PAGE and Western blot using an anti-myc monoclonal antibody. Equal amount of total protein was loaded into each well. (B) Quantitation by densitometry of the C-terminal fragment detected in *A.* Values represent the proportion of this myocilin fragment, expressed as indicated in Fig. 1B. (C) Densitometric quantitation of the full-length and C-terminal myocilin fragment detected in *A.* Error bars correspond to the SD of three independent experiments carried out in triplicate. Asterisks indicate statistical significance compared to control: p<0.01 (**); p<0.001 (***). One-way ANOVA followed by Tukey multiple-comparison test.

**Figure 8:**
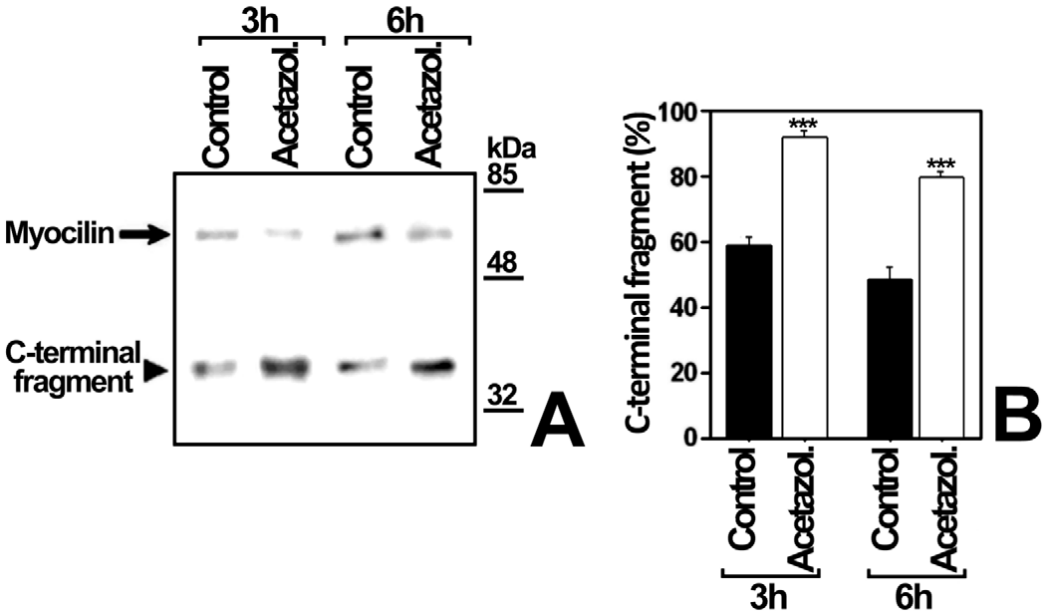
Effect of carbonic anhydrase inhibition on myocilin proteolytic processing. (A) HEK-293T cells (500000 cells/plate) were transfected with a cDNA construct encoding myocilin-myc. After transfection cells were cultured in bicarbonate-free HEPES buffered medium (75 mM, pH 7.1) for 3 or 6 h in the presence of acetazolamide (0.25 mM). Extracellular recombinant myocilin was analyzed by 10% polyacrylamide SDS-PAGE and Western blot using an anti-myc monoclonal antibody. Equal amount of total protein was loaded into each well. (B) Quantitation by densitometry of the C-terminal fragment detected in *A.* Values represent the proportion of this myocilin fragment, expressed as indicated in Fig. 1B. Error bars correspond to the SD of two independent experiments carried out in triplicate. Asterisks indicate one-way ANOVA statistical significance compared to control: p<0.001 (***).

**Figure 9:**
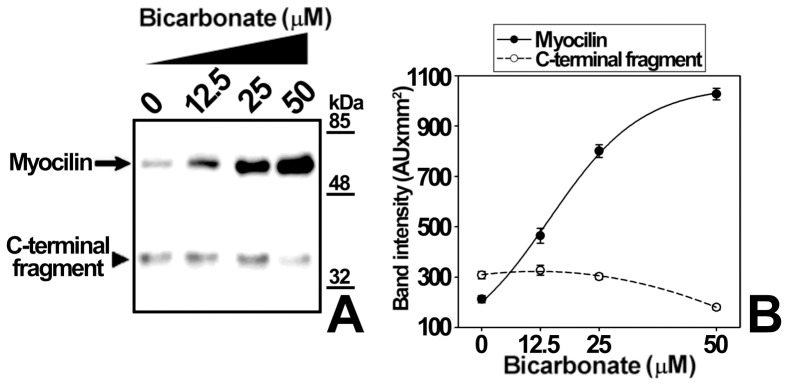
Effect of extracellular sodium bicarbonate on myocilin secretion and proteolytic processing. (A) HEK-293T cells (500000 cells/well) were transfected with a cDNA construct encoding myocilin-myc. After transfection cells were cultured in bicarbonate-free HEPES buffered medium (75 mM, pH 7.1) in the presence of different bicarbonate concentrations. Culture media were collected 3 hours later and extracellular recombinant myocilin was analyzed by 10% polyacrylamide SDS-PAGE and Western blot using an anti-myc monoclonal antibody. Equal amount of total protein was loaded into each well. (B) Quantitation by densitometry of the C-terminal fragment detected in *A.* Values represent the proportion of this myocilin fragment, expressed as indicated in Fig. 1B. Error bars correspond to the SD of three independent experiments carried out in triplicate. One-way ANOVA analysis for full-length myocilin and C-terminal fragment, p<0.001 and p<0.05, respectively.

**Figure 10:**
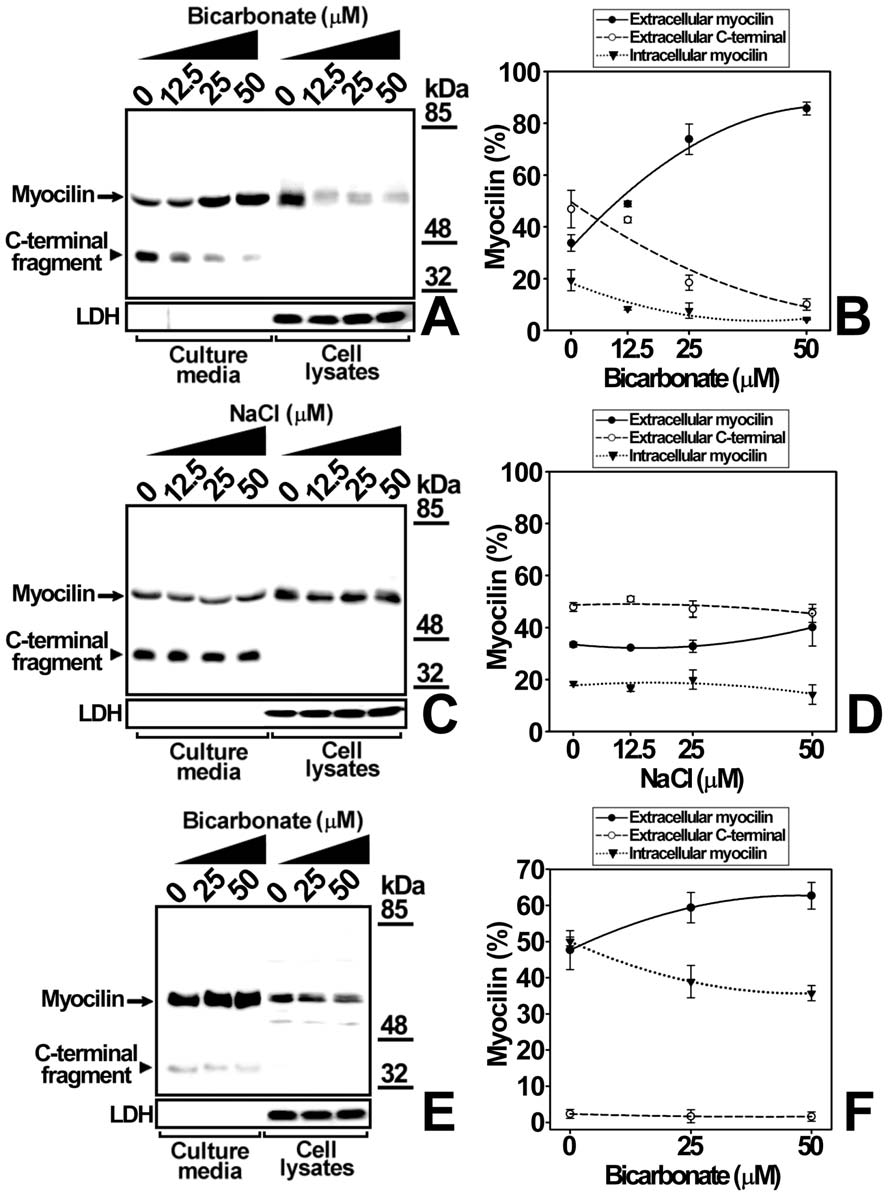
Specificity of extracellular bicarbonate effect on myocilin secretion. HEK-293T cells (400000 cells/well) were transfected with a cDNA construct encoding myocilin-myc. After transfection the cells were incubated in DMEM for 48 h with increasing concentrations of either sodium bicarbonate (A) or control NaCl (C). Recombinant myocilin was analyzed both in the culture medium and cells by 10% polyacrylamide SDS-PAGE and Western blot using an anti-myc monoclonal antibody. Cell lysates and culture medium samples contained 20 and 80 μg of total protein, respectively. As an internal control of sample loading or cell integrity LDH was detected in cell extracts and culture media, respectively, using a goat anti-LDH antibody. (B) and (D) quantitation by densitometry of recombinant myocilin detected in *A* and *C,* respectively. (E) The human ocular cell line hCM stably expressing recombinant myocilin-HA (400000 cells/well) was incubated with increasing concentrations of sodium bicarbonate as indicated in A. Recombinant myocilin and LDH were also analyzed as indicated in A using anti-HA and anti-LDH monoclonal antibodies, respectively. (F) Quantitation by densitometry of recombinant myocilin detected in *E.* Error bars correspond to the SD of three independent experiments carried out in triplicate. One-way ANOVA analysis for extracellular full-length myocilin, extracellular C-terminal fragment and intracellular myocilin in B, p<0.001, p<0.05 and p<0.01, respectively. One-way ANOVA analysis in D, p>0.5. One-way ANOVA analysis for extracellular full-length myocilin, extracellular C-terminal fragment and intracellular myocilin in F, p<0.05, p<0.01 and p>0.05, respectively.

To test if culture medium pH changes modulate myocilin proteolytic processing, cells transiently expressing recombinant myocilin were cultured at different pH values, obtained by addition of acids (ascorbic acid or N-acetyl-cysteine, which is also an antioxidant) or bases (NaOH, piruvate or bicarbonate) (Fig. 5A). Culture medium acidification led to a 3-fold increase in the extracellular proportion of the myocilin C-terminal fragment (Fig. 5B, Ascorbic Acid and N-Ac-Cys). In contrast, the culture medium alkalinization diminished processing from 1.5 to approximately 14 times (Fig. 5B, NaOH Piruvate and Bicarbonate). In order to control extracellular pH variations associated with CO_2_ concentration changes when cells are removed from the incubator, we repeated the experiment using bicarbonate-free medium, buffered with HEPES at pH values ranging from 6.2 to 8.2 (Fig. 6). Unexpectedly, at pH values from 6.9 to 7.8, almost only full-length myocilin was present extracellularly. No extracellular myocilin was detected at pH 6.2 and 8.2, due to reduced cell viability determined by a MTT assay (data not shown). The low extracellular C-terminal fragment proportion in bicarbonate free-media contrasts with its abundance in bicarbonate-buffered media, suggesting that this proportion depends on bicarbonate concentration variations rather than on pH changes. In addition, a time-course analysis (3–48 h) of extracellular recombinant myocilin, expressed in HEPES-buffered bicarbonate-free medium at pH 7.1, showed that the amount of the C-terminal fragment remained constant, but full-length myocilin increased over time (Fig. 7A and C), resulting in lower extracellular C-terminal fragment proportion (Fig. 7B). Based on these results, we hypothesized that bicarbonate produced by cellular carbonic anhydrases could be responsible for the increase in extracellular full-length myocilin. To test this hypothesis, recombinant myocilin was expressed either for 3 h or 6 h in HEPES-buffered culture medium, pH 7.1, supplemented with the carbonic anhydrase inhibitor acetazolamide. This treatment slightly reduced the presence of full-length myocilin, but significantly increased both the total amount and proportion of the extracellular C-terminal fragment (Fig. 8 and B). Furthermore, increasing bicarbonate concentrations in HEPES-buffered culture medium, pH 7.1, resulted in significantly increased extracellular full-length myocilin and decreased C-terminal fragment (p<0.001 and p = 0.002,respectively; one-way ANOVA) (Fig. 9A and B). To assess the possible effect of HEPES on these observations at the concentration used (75 mM), we expressed myocilin in HEK-293T cells grown in sodium bicarbonate-free culture media buffered at pH 7.1 with different concentrations of HEPES, ranging from 25 to 75 mM. Although we observed small variations of extracellular full-length myocilin and its C-terminal fragment the differences were not significant (p>0.2; one-way ANOVA) (Fig. S2), indicating that at this concentration HEPES does not influence neither myocilin secretion nor cleavage. The effect of sodium bicarbonate on both extracellar full-length myocilin and C-terminal fragment was also replicated in bicarbonate-buffered media (Fig. 10A and B). In this assay intracellular myocilin was analyzed and we observed that it decreased (p>0.05; one-way ANOVA) as the extracellular protein increased (p>0.05; one-way ANOVA) (Fig. 10A and B). As a control cells were cultured in parallel with increasing NaCl concentrations (12.5–50 μM). Both myocilin secretion and processing were unaffected by this treatment (Fig. 10C and D), indicating that they are not influenced by sodium or osmolarity variations, and further supporting that these effects are specifically mediated by bicarbonate. Finally, we used the ocular cell line hCM stably expressing recombinant myocilin to replicate the results. Although it is known that this cell line processes myocilin less efficiently than HEK-293T cells [9], we also observed a significant bicarbonate-dependent increased secretion of full-length myocilin (p = 0,01); one-way ANOVA) along with a parallel decrease of the intracellular protein (p = 0.03; one-way ANOVA) (Figs. 10E and F). These data show that the extracellular concentration of bicarbonate influences the presence of extracellular full-length recombinant myocilin both in non ocular and ocular cell lines.

### Bicarbonate-dependent Extracellular C-terminal Fragment Proportion

**Figure 11:**
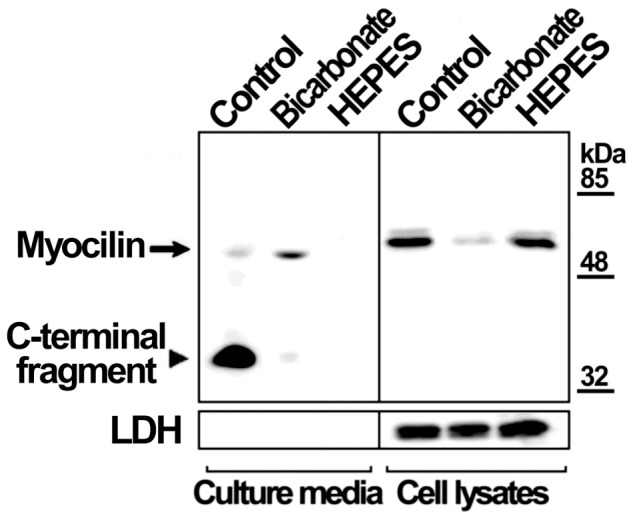
Effect of extracellular bicarbonate on full-length myocilin secretion. HEK-293T cells (500000 cells/well in 24-well plates) were transfected with a cDNA construct encoding myocilin-myc. After transfection cells were cultured in 200 ul of bicarbonate buffered medium for 39 h (control). Other cells were cultured in the same medium for 36 h, then the culture medium was removed and the cells were incubated with either fresh bicarbonate-buffered medium (bicarbonate) or HEPES-buffered medium (75 mM, pH 7.1) (HEPES) for 3 h. Cell lysates samples contained 20 μg of total protein. Bicarbonate-buffered medium and HEPES-buffered medium aliquots contained 80 and 2 μg of total protein, respectively. Extracellular and intracellular recombinant myocilin and LDH were analyzed by 10% polyacrylamide SDS-PAGE and Western blot using anti-myc or anti LDH monoclonal antibodies, respectively. The experiment was repeated three times independently.

To further support the effect of bicarbonate on extracellular C-terminal fragment proportion, myocilin transfected HEK-293T cells were incubated with bicarbonate-buffered medium for 36 h. Then, as a control, culture medium and cells were collected (Fig. 11, Control). Treated cells were further incubated for 3 h with either bicarbonate-buffered medium, pH 7.4 (Fig. 11, Bicarbonate), or fresh HEPES-buffered culture medium, pH 7.1 (Fig. 11, HEPES) and the presence of recombinant myocilin in the culture media and cells was analyzed by SDS-PAGE and Western blot. Control cells exhibited an extracellular C-terminal fragment percentage higher than 95% (Fig. 11, Culture media, Control) along with a significant amount of intracellular full-length myocilin (Fig. 11, Cell lysates, Control). Cells treated with fresh bicarbonate-buffered medium increased the presence of extracellular full-length myocilin (Fig. 11, Culture media, Bicarbonate), but in parallel decreased intracellular full-length myocilin (Fig. 11, Cell lysates, Bicarbonate). Conversely, the cells incubated with fresh bicarbonate-free HEPES-buffered medium only presented intracellular full-length myocilin (Fig. 11, Culture media and Cell lysates, HEPES). These data strongly support that extracellular bicarbonate promotes the secretion of full-length myocilin and that the exhaustion of this anion leads to an intracellular accumulation of the protein.

### Identification of the Myocilin Region Involved in its Bicarbonate-dependent Intracellular Retention

**Figure 12:**
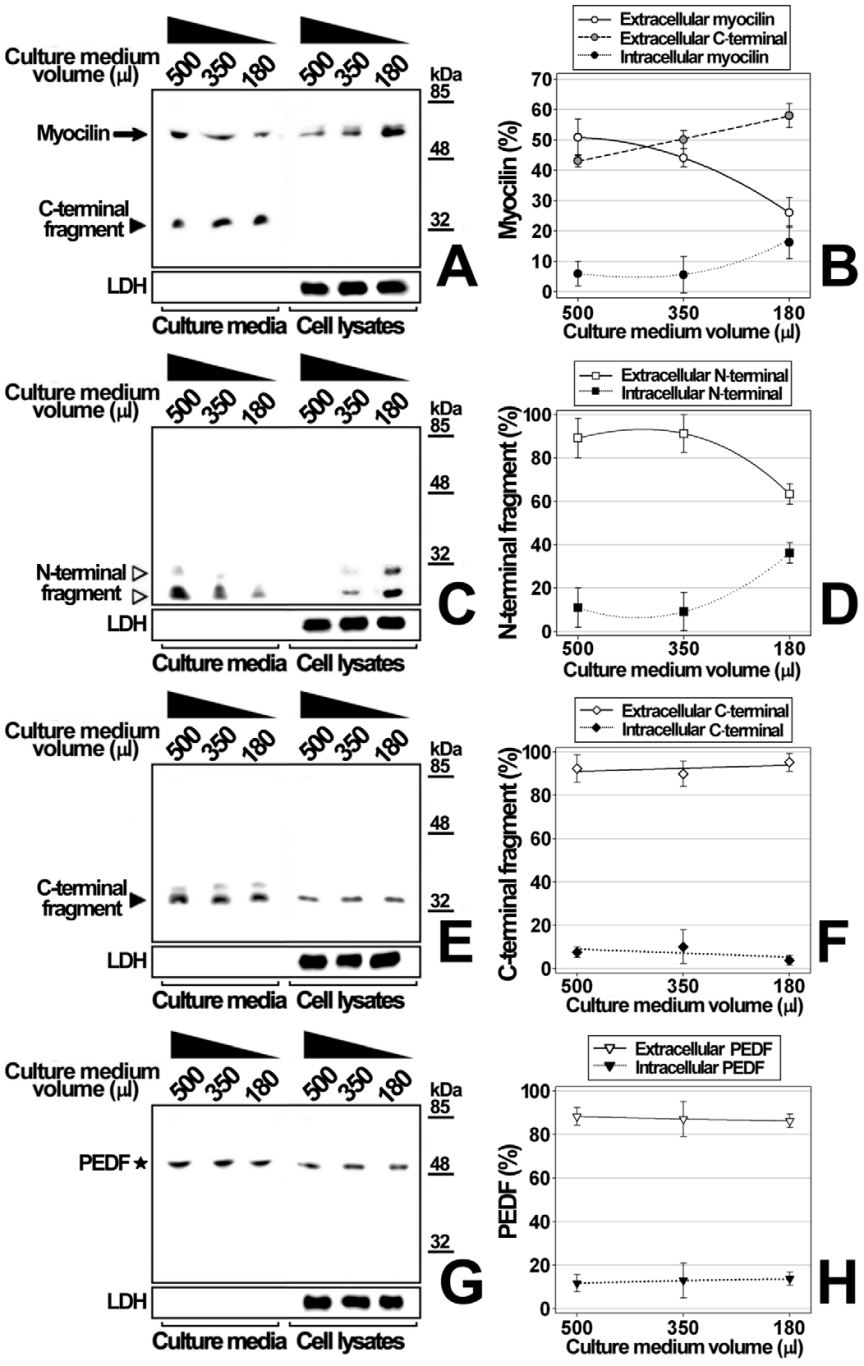
Identification of the myocilin region involved in its intracellular accumulation in response to extracellular bicarbonate-depletion. HEK-293T cells (400000 cells/plate in 24-well plates) were transfected with cDNA constructs encoding full-length myocilin (A), a myocilin N-terminal fragment (amino acids 1–226) (C), a myocilin C-terminal fragment (amino acids 217–504) (E) or the control protein PEDF (G). All the recombinant proteins were fused to the myc epitope on their C-terminal ends. After transfection, cells were incubated for 48 h in 3 different culture medium volumes (500, 350 and 180 ul). Extracellular and intracellular recombinant proteins were analyzed in aliquots representing 5% of either the total culture medium volume or cell lysate, by 10% polyacrylamide SDS-PAGE and Western blot using an anti-myc monoclonal antibody. As an internal control of sample loading or cell integrity LDH was detected in cell extracts and culture media, respectively, using a goat anti-LDH antibody. (B, D, F and H). Quantitation by densitometry of the recombinant proteins detected in *A, C, E, and G,* respectively. Error bars correspond to the SD of three independent experiments carried out in triplicate. One-way ANOVA analysis for extracellular full-length myocilin, extracellular C-terminal fragment and intracellular myocilin in B, p<0.01, p<0.01 and p = 0.05, respectively. One-way ANOVA analysis for extracellular and intracellular N-terminal fragment in panel D, p<0.01 and p<0.001, respectively. One-way ANOVA analysis in F and H, p>0.05.

In order to analyze the possible role of the two myocilin regions in the intracellular accumulation of the protein observed in bicarbonate-depleted media, we analyzed the cellular distribution of the full-length protein and its recombinant N- and C-terminal fragments, independently expressed in HEK293T cells. Extracellular bicarbonate-depletion was metabolically induced by culturing cells at decreasing culture medium volumes (500, 350 and 180 ul) for 48 h (Fig. 12). As expected, intracellular full-length myocilin increased, and in parallel the extracellular protein decreased inversely proportional to the culture medium volume reduction (Fig. 12A and B). Interestingly, this intracellular accumulation was associated with a moderately increased extracellular C-terminal fragment (Fig. 12A and B), suggesting a connection between the intracellular retention of the protein and its proteolytic cleavage. The recombinant N-terminal fragment showed the same behaviour as the full-length protein (Fig. 12C and D), whereas the distribution of both the recombinant C-terminal fragment and control protein PEDF was unaltered by these treatments (Fig. 12E–H), showing the specificity of the phenomenon. These results show that myocilin intracellular retention induced by extracellular bicarbonate-exhaustion is mediated by its N-terminal domain, and that the retention boosts myocilin proteolysis.

## Discussion

The proteolytic processing of myocilin was identified *in vitro* by our group [9] and we also suggested the existence of culture medium factors that might regulate recombinant myocilin proteolytic cleavage [10]. Myocilin fragments have also been detected in different tissues, supporting that they are also produced physiologically [9], [14]. Proteolytic cleavage plays an important role in regulating the function of different proteins, including proteolytic enzymes, protein hormones and neuropeptides [19]. On the other hand, culture conditions have been described to affect cellular processes such as trafficking of proteolytic enzymes [20]. Therefore, and as an approach to unravel the enigmatic biological function of this protein, the main goal of this study was to identify the possible factors regulating the proteolytic processing of recombinant myocilin.

The proteolytic cleavage of the protein was experimentally estimated as the extracellular C-terminal fragment proportion. We determined that this percentage is not only directly proportional to two dependent variables i.e., initial cell density and culture time, but inversely proportional to the culture medium volume. These parameters change the chemical composition of the culture medium. Thus, we hypothesized that an extracellular metabolite could cause this effect. Oxidizing agents and free radicals were considered candidate compounds because they accumulate over time in the culture medium. Nevertheless, our results indicated that oxidizing agents do not play a direct role in the proteolytic cleavage of myocilin. Then, we studied the role of metabolic-induced culture medium acidification, and we observed that the extracellular C-terminal fragment percentage raised at low pH values in bicarbonate-buffered culture media. Unexpectedly, this ratio remained constant at different pH values in bicarbonate-free HEPES-buffered medium, showing that this ratio is pH-independent. Therefore, we speculated that the metabolic-induced depletion of this anion in bicarbonate-buffered media could lead to a relative C-terminal fragment increase, as a result of extracellular full-length myocilin reduction. In other words, these data indicate that the amount of extracellular full-length myocilin is directly proportional to the extracellular concentration of bicarbonate. This hypothesis was supported by two observations: first, bicarbonate induced a dose-dependent extracellular full-length myocilin increase; second, in bicarbonate-free medium, the amount of full-length extracellular myocilin increased over time due to endogenous bicarbonate production. Therefore, metabolic differences among cell lines lead to different bicarbonate-depletion rates, which potentially explain the distinct C-terminal fragment proportion produced by different cell lines [9], [18]. Our data also suggest that extracellular bicarbonate depletion also favours the proteolytic processing of the protein, as it leads to the intracellular accumulation of full-length myocilin, thus facilitating proteolysis in the ER lumen by Calpain II [10]. In fact, we observed that the extracellular C-terminal fragment increased when full-length myocilin was intracellularly retained (Fig. 3, 12A and B).

We also conclude that myocilin’s N-terminal domain is involved in the intracellular retention and secretion of the protein, in accordance with previous reports [10]. In line with this idea, myocilin has been reported to be secreted via a non conventional exosome-mediated pathway [21] in which the N-terminal coiled-coil domain mediates myocilin binding to secretory vesicles [22]. This myocilin region contains a leucine-zipper motif [4], homologous to soluble NSF attachment protein receptors (SNAREs) and involved in the formation of a membrane-associated complex with hydrodynamic properties similar to those of known SNARE complexes [23]. SNARE molecules mediate membrane fusion in the secretory pathway of eukaryotic cells. Interestingly, and according to our results, a bicarbonate-dependent interaction between complexin 1/2 and the SNARE complex in acrosomal exocytosis has been suggested [24]. Therefore, we hypothesize that the observed bicarbonate-dependent intracellular accumulation of myocilin could be due to interactions with SNARE molecules. However, much remains to be investigated as regards the biological relevance of these interactions.

These results may also have potential pathophysiological implications. Experimental intraocular pressure (IOP) increment is associated with acidification of ocular tissues in cat retinas [25] and rabbit vitreous [26]. Therefore according to our data, one could reasonably infer that IOP rising could, at least transiently, diminish AH bicarbonate concentration, inducing intracellular myocilin retention and proteolytic cleavage in ocular tissues such as ciliary body and trabecular meshwork. This mechanism might finally decrease myocilin homo- and hetero-aggregates, contributing to lower IOP, as previously suggested [14], [15]. Besides, we have shown that *MYOC* mutations reduce the proteolytic processing of recombinant myocilin [13] and they have been recently shown to also up-regulate Cystatin A expression, a cysteine protease inhibitor that has been proposed to potentially participate in the inhibition of the process [18].

Although these hypotheses await to be confirmed, identifying bicarbonate as a factor that regulates recombinant myocilin secretion and proteolytic cleavage, might open up new avenues to understand the biological function of this protein in the eye and in glaucoma pathogenesis.

## Supporting Information

Figure S1 **Pre-conditionated culture medium influence on myocilin proteolytic processing. (A)** HEK-293T cells (400000 cells/plate) were transfected with a cDNA construct encoding myocilin-myc. After transfection, culture medium (500 µl) pre-incubated during 24 h with the indicated non transfected HEK-293T conditioning cell number (CCN) was added to each well and collected 48 hours later. Extracellular recombinant myocilin was analyzed by 10% poliacrylamide SDS-PAGE and western blot using an anti-myc monoclonal antibody. Equal amount of total protein was loaded into each well. **(B)** Densitometric relative quantitation of the C-terminal fragment detected in *A.* Please note that the Y-axis scale has been selected to facilitate visualization of differences between samples. Error bars correspond to SD of triplicate experiments. Asterisks indicate statistical significance compared to 0 CCN: p<0.001 (***), one-way ANOVA followed by Tukey multiple-comparison test.
(TIF)

Figure S2 **Effect of HEPES concentration on myocilin proteolytic processing. (A)** HEK-293T cells (500000 cells/plate) were transfected with a cDNA construct encoding myocilin-myc. After transfection cells were cultured in bicarbonate-free medium in the presence of increasing HEPES pH7.1 concentrations (25–75 mM). Culture media were collected 48 hours later and extracellular recombinant myocilin was analyzed by 10% polyacrylamide SDS-PAGE and Western blot using an anti-myc monoclonal antibody. Equal amount of total protein was loaded into each well. **(B)** Densitometric quantitation of the full-length and C-terminal myocilin fragment detected in *A.* Error bars correspond to the SD of three independent experiments carried out in triplicate. One-way ANOVA analysis did not show significant differences (p>0.05).
(TIF)
