# Supplementary figures and images for: Bicarbonate-Dependent Secretion and Proteolytic Processing of Recombinant Myocilin
Source: PLoS One. 2013 Jan 16;8(1):e54385. doi: 10.1371/journal.pone.0054385 (PMC3547000; doi:10.1371/journal.pone.0054385)

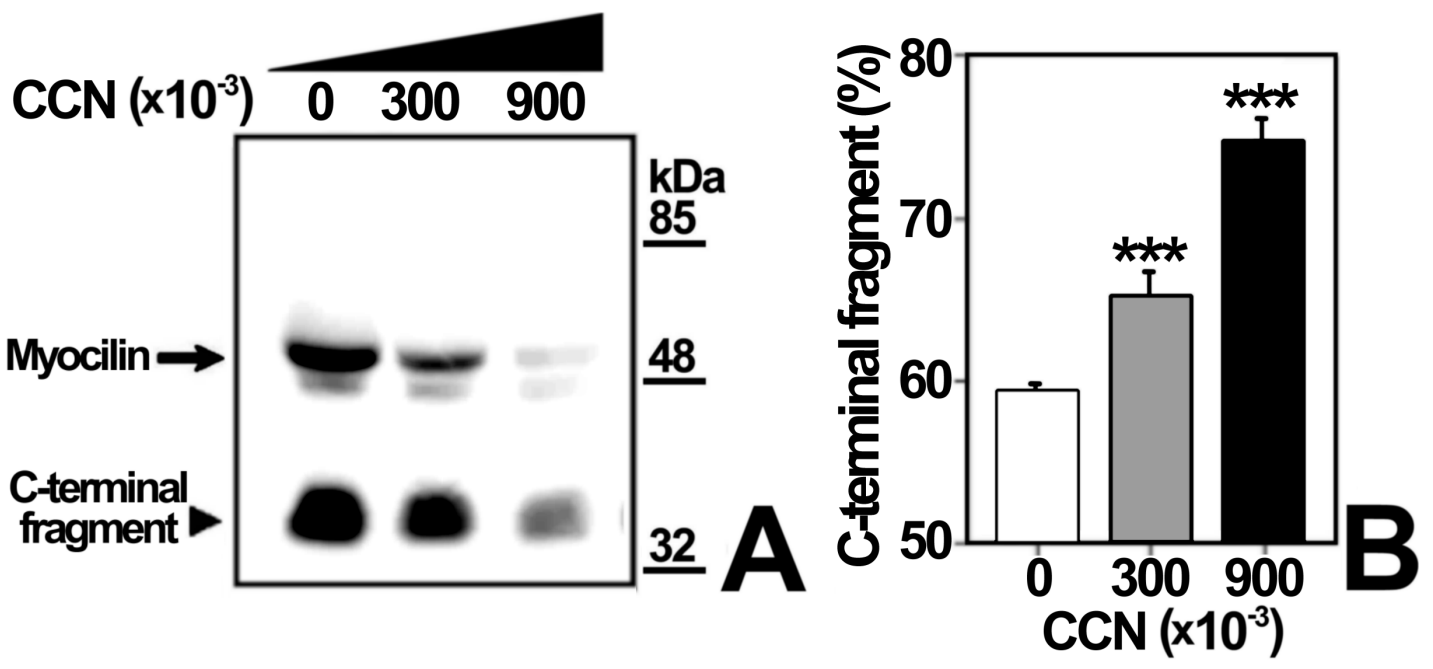

Supplement: Figure S1 — Pre-conditionated culture medium influence on myocilin proteolytic processing. (A) HEK-293T cells (400000 cells/plate) were transfected with a cDNA construct encoding myocilin-myc. After transfection, culture medium (500 µl) pre-incubated during 24 h with the indicated non transfected HEK-293T conditioning cell number (CCN) was added to each well and collected 48 hours later. Extracellular recombinant myocilin was analyzed by 10% poliacrylamide SDS-PAGE and western blot using an anti-myc monoclonal antibody. Equal amount of total protein was loaded into each well. (B) Densitometric relative quantitation of the C-terminal fragment detected in A. Please note that the Y-axis scale has been selected to facilitate visualization of differences between samples. Error bars correspond to SD of triplicate experiments. Asterisks indicate statistical significance compared to 0 CCN: p<0.001 (***), one-way ANOVA followed by Tukey multiple-comparison test. (TIF) [file pone.0054385.s001.tif]

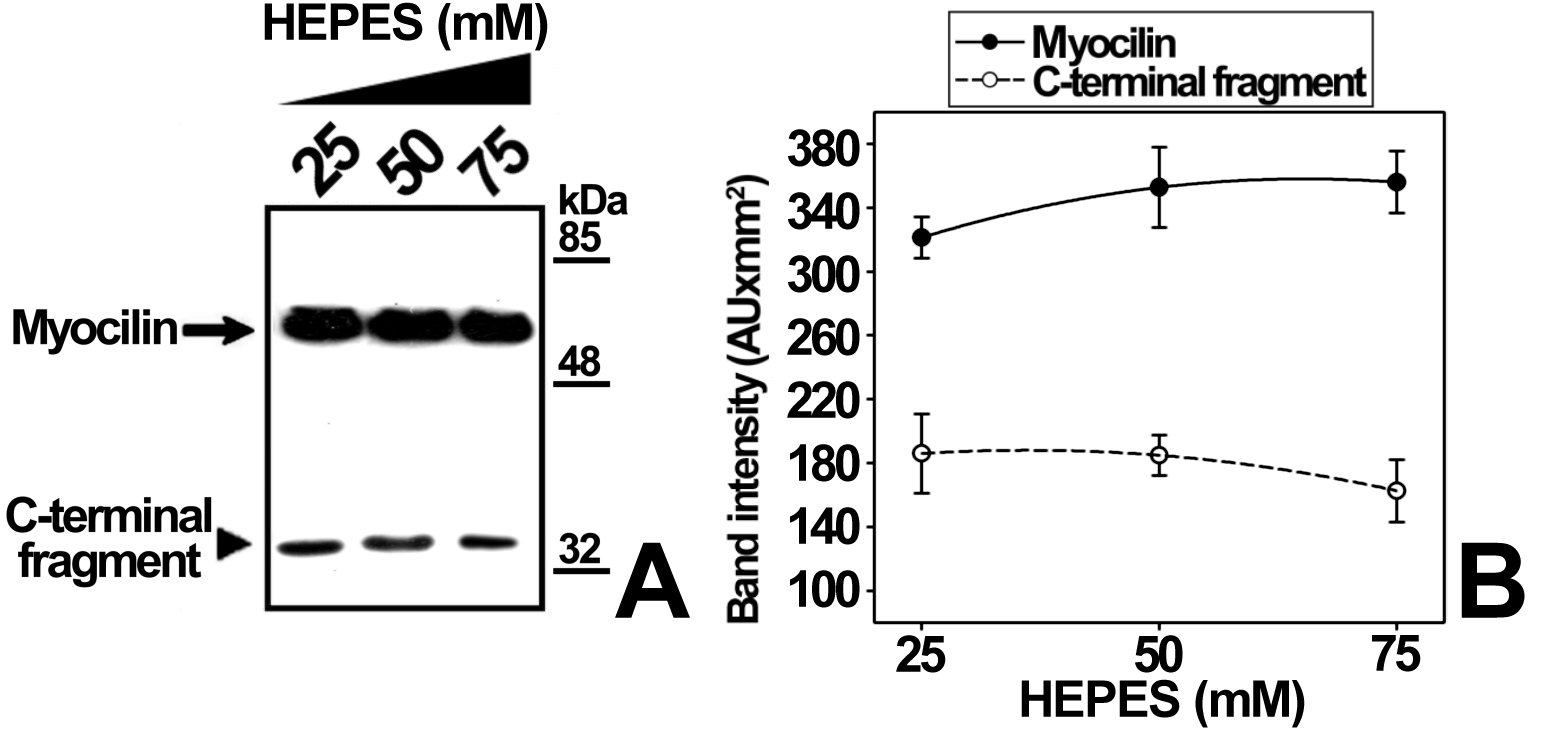

Supplement: Figure S2 — Effect of HEPES concentration on myocilin proteolytic processing. (A) HEK-293T cells (500000 cells/plate) were transfected with a cDNA construct encoding myocilin-myc. After transfection cells were cultured in bicarbonate-free medium in the presence of increasing HEPES pH7.1 concentrations (25–75 mM). Culture media were collected 48 hours later and extracellular recombinant myocilin was analyzed by 10% polyacrylamide SDS-PAGE and Western blot using an anti-myc monoclonal antibody. Equal amount of total protein was loaded into each well. (B) Densitometric quantitation of the full-length and C-terminal myocilin fragment detected in A. Error bars correspond to the SD of three independent experiments carried out in triplicate. One-way ANOVA analysis did not show significant differences (p>0.05). (TIF) [file pone.0054385.s002.tif]
